# The Impact of Recombinant Versus Plasma-Derived Factor VIII Concentrates on Inhibitor Development in Previously Untreated Patients With Hemophilia A: A 2021 Update of a Systematic Review and Meta-Analysis

**DOI:** 10.7759/cureus.26015

**Published:** 2022-06-16

**Authors:** Kelvin Kohar, Stephanie A Prayogo, Lowilius Wiyono

**Affiliations:** 1 Faculty of Medicine, Universitas Indonesia, Jakarta, IDN

**Keywords:** meta-analysis, systematic review, inhibitor, plasma-derived factor viii, recombinant factor fviii, hemophilia a

## Abstract

Hemophilia A, the most common hereditary disorder, is caused by clotting factor deficiency. Challenges encountered in the current treatment of hemophilia A [factor VIII (FVIII) replacement therapy] due to inhibitor development have caused ineffective treatment as well as morbidity and mortality among patients. However, there are no studies comparing the two types of FVIII treatments in terms of inhibitor development rate. Therefore, we conducted this systematic review to devise a better treatment option with a lower risk of inhibitor development. The systematic review was conducted using Preferred Reporting Items for Systematic Reviews and Meta-Analyses (PRISMA) guidelines and by searching several databases. Data extraction on study characteristics and outcomes was conducted. Reviewers also conducted a risk of bias assessment on all studies. All eligible studies for quantitative analysis were then processed using RevMan 5.4.1 and the data was extrapolated into cumulative outcomes and expressed in forest and funnel plots. Nine studies were included in the meta-analysis, involving a total of 2,531 hemophilia A patients who were followed up from birth until death. A higher incidence of inhibitor development was found to be associated with recombinant FVIII (rFVIII) [odds ratio (OR)=1.57, 95% confidence interval (CI): 0.95-2.59; hazard ratio (HR)=1.89, 95% CI: 1.15-3.12]. The same trend was also found for high-responding inhibitors (OR=1.38, 95% CI: 0.70-2.70; HR=1.42, 95% CI: 0.84-2.39). rFVIII is associated with a higher risk of overall and high-responding inhibitor development compared to plasma-derived FVIII (pdFVIII).

## Introduction and background

Hemophilia is a genetic condition causing prolonged bleeding and may be difficult to control due to inadequate clotting factors, factor VIII (FVIII) or factor IX (FIX), which are needed for blood clot regulation. Internal bleeding remains the main problem in patients with hemophilia. Bleeding or hemorrhage can occur in joints, such as elbows, ankles, and knees. This may be the result of an injury, but it may also occur voluntarily in acute hemophilia. Hemophilia is a rare inherited bleeding disorder and can be found in one out of 3,333 people [1].

The diagnosis and treatment methods of hemophilia vary greatly around the world, and mainly reflect the socioeconomic status of a country. In low-income countries, most patients are underdiagnosed, resulting in patients dying prematurely due to a lack of treatment [2]. Hemophilia is mainly classified into three groups: hemophilia A, hemophilia B, and hemophilia C. Hemophilia A, B, and C result from deficiency or dysfunction of FVIII, FIX, and FXI, respectively. Hemophilia A is the most common of the three types, comprising 80-85% of total cases. On the other hand, hemophilia C is rarely found. As it is an X-linked recessive genetic disease, it commonly affects males [3]. In rare cases, this disease can also be acquired. Acquired hemophilia is caused by autoantibodies that develop against a coagulation factor [4].

The current treatment available for hemophilia A involves replacing the missing clotting factor (FVIII) by way of intravenous transfusion. This is usually done by injecting a therapeutic product, called clotting FVIII concentrates, which are either plasma-derived (pdFVIII) or recombinant (rFVIII) [5]. Clotting factor replacement therapy usage has greatly advanced in developing countries, facilitated by a modern healthcare system that provides generous funding for the supply of clotting factors [6]. However, the replacement therapy can induce inhibitor development. Inhibitors develop in 30% of patients with hemophilia A on replacement therapy [7]. Besides the clotting factor replacement therapy, aspects such as gene mutations, age, and family history can also be risk factors for inhibitor development. Inhibitor development occurs when antibodies against “foreign” FVIII are produced, leading to the low efficacy of such treatment. People with anti-FVIII antibodies can be managed with immune tolerance induction (ITI) with 60-80% effectiveness. However, ITI is costly and requires a regular infusion of FVIII for months to years, and is sometimes even unsuccessful. Moreover, inhibitor recurrence is also reported in quite a fair proportion of cases [8].

For many years, immune tolerance to factors has been a major concern, since the development of inhibitors will lower the quality of life and significantly increase morbidity and mortality among patients [9]. The type of replacement therapy, as one of the risk factors of inhibitor development (plasma-derived vs. recombinant), received by patients can be improved to minimize the risk. Choosing the right type of replacement therapy (plasma-derived vs. recombinant) can be a way to reduce the probability of inhibitor development.

Even though systematic reviews have already been conducted on this topic in 2010 and 2012, the significance of the results was not then demonstrated. Moreover, they only included one-arm studies, which resulted in ambiguous data and remain inconclusive. As more studies were conducted during the period 2012-2021, an updated systematic review and meta-analysis is needed to address the question of whether plasma-derived or recombinant FVIII (pdFVIII or rFVIII) results in increased inhibitor development and to engage in a comparative analysis of the two in clinical studies [10,11].

## Review

Materials and methods

The systematic review was conducted using the Preferred Reporting Items for Systematic Review and Meta-Analysis (PRISMA) guidelines by objectively performing a search and screening process on relevant studies on the topic [12]. The complete protocol for the systematic review has been previously registered in PROSPERO (CRD42021248188).

Search Strategy

A literature search was conducted in a blinded fashion by two independent investigators (SAP, KK). Any discrepancies were resolved by discussion among the authors (SAP, KK, LW). The literature search was conducted on several scientific databases, such as PubMed, Scopus, and ScienceDirect, using predetermined keywords and medical subject headings (MeSH) on hemophilia A, pdFVIII, rFVIII, and inhibitors. Search strategy and Boolean Operators used on each scientific database are mentioned in Table 3 in the appendices. Subsequently, the retrieved search results were manually deduplicated and screened using the pre-determined eligibility criteria.

Study Eligibility Criteria

Screening processes were then used to filter all articles found in three different databases. The initial yield of the searching process was 595 articles. All articles were then filtered using predetermined inclusion and exclusion criteria, based on the PICO (Patient, Intervention, Comparison, Outcome) criteria in hemophilia A patients who use pdFVIII or rFVIII, and the incidence rate of inhibitor development as its outcome. The included studies consisted of prospective and retrospective cohort studies, randomized controlled trials (RCTs), and case-control studies that evaluated the comparison of pdFVIII and rFVIII on their inhibitor incidence rate. Studies were excluded based on the following criteria: (1) review articles, case series or case reports, and letters to editors; (2) animal studies (non-human clinical studies); (3) inaccessible or irretrievable full-text articles; (4) non-English articles; (5) articles published more than 10 years ago (before 2011). A limitation on publication date was also applied so as to avoid including studies that have been used in the previous cumulative analysis on similar topics.

Study Selection

The search and screening process of all studies was conducted using Google Sheet (Google LLC, Mountain View, CA). The articles were then deduplicated to remove duplicates. All articles were then independently reviewed by each investigator based on the PRISMA guideline scheme. The screening process started with the title and abstract screening of the selected articles to exclude studies based on the exclusion criteria. The investigators then documented the underlying reason for exclusion in the spreadsheet. Studies were included in the next step if there was uncertainty or if their exclusion was disputed by any of the investigators. Afterward, all investigators independently read the full texts of all selected studies to exclude studies that met any of the exclusion criteria. All selected studies were then validated by all reviewers to take a final decision on the eligible studies to be included for qualitative and quantitative analysis.

Data Extraction and Quality Assessment

Data extraction was conducted using a predetermined form with Google Sheet (Google LLC). All investigators extracted the data from each eligible article independently. The following data were extracted from each study: study authors, publication year, study design, study location (geographical), sample size, patient characteristics, patients' median age, and the primary endpoint of the study. Investigators also recorded outcomes, especially inhibitor incidence rates, high-responding inhibitor incidence, and low-responding inhibitor incidence, and reported the [odds ratio (OR)/hazard ratio (HR)] value of each study.

The eligible studies were then assessed for methodological quality assessment in order to minimize systematic biases and inferential errors from the extracted data. All reviewers independently assessed the risk of bias in the included cohort and case-control studies using the Newcastle-Ottawa Quality Assessment Scale (NOS) [13]. The NOS risk of bias tool evaluates non-randomized studies on systematic reviews on three quality parameters: study selection, comparability of the population, and a determination of whether the exposure or outcome includes a risk of bias [14]. NOS evaluated each study's quality and yielded a maximum score of 9 points. Studies with NOS scores greater than or equal to 7 were considered high quality. Studies that scored 5 and 6 were considered fair or moderate quality, whereas studies having NOS scores of less than 5 points indicated a high risk of bias [14]. Meanwhile, the RCT study was assessed using the risk of bias (RoB 2) tool by Cochrane. The tool assesses RCTs on bias arising from randomization processes, deviations from intended interventions, missing outcome data, measurement of outcome, and selection of reported results [15].

Pooled Analysis

The pooled analysis was conducted using Review Manager (RevMan) 5.4 (The Cochrane Collaboration, 2020). The cumulative incidence of inhibitors was evaluated in each study and then categorized within groups. These were identified using rFVIII and pdFVIII. No further categorization of each FVIII product was conducted to assess all types of FVIII products. Cumulative incidences were then categorized into high-responding inhibitors and low-responding inhibitors, entailing the response to FVIII use on the inhibitor levels found in the patient. The classification was made based on the available comparison made by the included studies, to properly distinguish the inhibitor development rate difference between low-responding and high-responding inhibitors.

Summary data and related 95% confidence intervals (CI) were then calculated by conventional meta-analysis pooling on logits [ln(odds)] from each individual study. Quantitative analysis was done using random effects-inverse variance, whereas logits were converted to rates, and data were reported in OR. Studies that mentioned HR values were also subjected to cumulative analysis to report cumulative HR values. Analysis of high-responding inhibitor rates was also conducted. All results were then visualized using forest plots and funnel plots. The indexes of heterogeneity (X^2^ or Q according to Cochran, I^2^, and tau^2^) were also calculated to analyze data distribution in each study [16].

Results

Study Selection, Study Characteristics, and Quality Assessment

**Figure 1:**
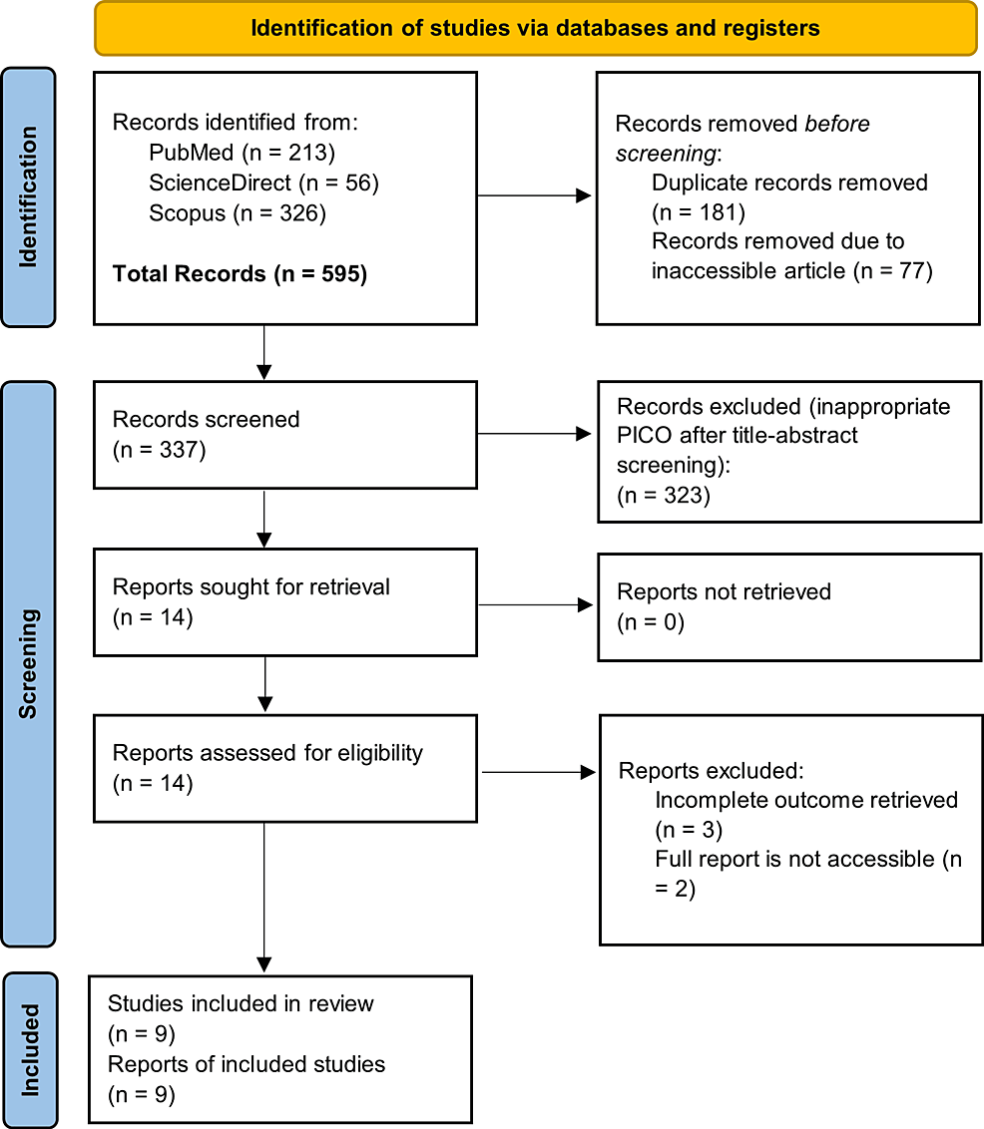
PRISMA flowchart on study selection PRISMA: Preferred Reporting Items for Systematic Reviews and Meta-Analyses

The authors obtained a total of 595 studies upon initial search. After removing 337 duplicates, the authors performed titles and/or abstracts screening and found 14 articles that would be assessed afterward at the full-text level. We further excluded five studies (of which three had ineligible data and two had inaccessible full text). Ultimately, nine articles were included in this systematic review. The selection process is described in Figure 1.

All included studies (seven cohorts, one case-control, and one randomized trial) were qualitatively and quantitatively synthesized [17-25]. The studies were performed in hemophilia centers in various countries; four were conducted in multiple countries [17,18,20,23], three in Europe [19,21,22], and two in Asia [24,25]. The articles included were published between 2011 and 2021, and the provided data were collected between 1980 and 2015 with follow-up duration varying across studies. Among the total 2,531 included participants, the majority were children (<18 years old) followed during the study period from birth, the first therapy received, until death. Besides, the authors also included all hemophilia A types based on FVIII (FVIII blood-clotting protein), including severe and non-severe. Severe hemophilia A was defined as an FVIII concentration of less than 1% of the normal level. Patients with FVIII concentrations of more than 1% of normal levels were classified as non-severe hemophilia A cases.

The participants were then divided into two groups: those receiving rFVIII or pdFVIII products. rFVIII could be further divided into first, second, third, and fourth generations. However, only three studies reported the exact rFVIII products administered clearly. 

Each study was also assessed for its quality by using quality assessment tools: NOS for cohort studies (Table 4) and case-control studies (Table 5) and Cochrane RoB 2 tool for RCTs (Table 6), as depicted in the Appendices. In general, all studies were categorized as good quality and with a low risk of bias.

Study Outcomes

The summary of all nine studies' outcomes is shown in Table 1 and Table 2. The major outcome presented in this table involves inhibitor development. Firstly, for the overall inhibitor development, the ratio of inhibitor development in both groups was obtained. Afterward, more specific data about inhibitor development response, e.g., high-responding or low-responding inhibitors, were collected if available. These data were used to calculate the OR in overall and high-responding inhibitors. Subsequently, the authors also included the overall HR in rFVIII compared to pdFVIII data along with 95% CI and p-values provided by the studies.

**Table 1:** Baseline characteristics of included studies ^a^Only hemophilia A included rFVIII: recombinant factor VIII; pdFVIII: plasma-derived factor VIII; R: recombinant; PD: plasma-derived; NA: not available; FG: first generation; SG: second generation; TG: third generation

No.	Author; year	Recruitment period	Study characteristics						
			Country/region	Study design	Patient characteristics	Sample size (n)	Age, mean (range)	Group I	Group II
1	Blatny et al.; 2021 [17]	2005–2015	Central and Eastern Europe (7 countries)	Prospective cohort	Children with severe hemophilia A	144	10 (7-14) years	121 patients in rFVIII	23 patients (16%) in pdFVIII
2	van Velzen et al.; 2020 [18]	1980-2011	33 European centers and 1 Australian center	Case-control	Non-severe hemophilia A	298	23 (5-44) years	52 in FG; 45 in SG; 7 in TG	179 in pdFVIII
3	Calvez et al.; 2018 [19]	1994-2016	France	Prospective cohort	Children with hemophilia A	395	NA	127 in SG; 137 in TG	131 in pdFVIII
4	Pevyandi et al.; 2018 [20]	2010-2014	14 countries (SIPPET)	Randomized trial	Children <6 years with severe hemophilia	251	3.19 (1.03-9.91) years	126 in rFVIII	125 in pdFVIII
5	Batorova et al.; 2016 [21]	1997-2008	Slovakia	Prospective cohort	Hemophilia A	59	12.5 (4.5-12.5) years	9 in rFVIII	50 in pdFVIII
6	Blatny et al.; 2015 [22]	2003-2013	Czech Republic	Prospective cohort	Hemophilia A	96	3 years in rFVIII vs. 5 years in pdFVIII	45 in rFVIII	41 in pdFVIII
7	Xuan et al.; 2014 [23]	2002-2012	China	Prospective cohort	Hemophilia A and hemophilia B^a^	235	NA	132 in rFVIII	203 in pdFVIII
8	Gouw et al.; 2013 [24]	2000-2010	29 hemophilia centers	Prospective cohort	Severe hemophilia A	574	6.4 (4.0-8.9) years	157 in TG; 260 in SG; 59 in FG	88 in pdFVIII
9	Strauss et al.; 2011 [25]	1984-2008	Israel	Prospective cohort	Hemophilia A	479	30 (18-75) months in rFVIII vs. 60 (36-none) months in pdFVIII	43 in rFVIII	249 in pdFVIII

**Table 2:** Outcomes of included studies rFVIII: recombinant factor VIII; pdFVIII: plasma-derived factor VIII; R: recombinant; PD: plasma-derived; NA: not available

No.	Author; year	Group	Inhibitor development				
			Overall	High-responding inhibitors	Low-responding inhibitors	Overall hazard ratio (95% CI; p-value)	High-responding inhibitors hazard ratio
1	Blatny et al.; 2021 [17]	rFVIII (121) vs. pdFVIII (23)	20/121 in R vs. 5/23 in PD	13/121 in R vs. 3/23 in PD	NA	1.56 (0.24-10.06; p=0.64)	0.85 (0.24-2.99; p=0.80)
2	van Velzen et al.; 2020 [18]	rFVIII (119) vs. pdFVIII (179)	36/119 in R vs. 39/179 in PD	NA	NA	NA	NA
3	Calvez et al.; 2018 [19]	rFVIII (264) vs. pdFVIII (131)	96/264 in R vs. 25/131 in PD	56/264 in R vs. 14/131 in PD	40/264 in R vs. 11 in PD	1.41 (0.83-2.38; p=0.21)	1.64 (0.82-3.25; p=0.16)
4	Pevyandi et al.; 2018 [20]	rFVIII (264) vs. pdFVIII (131)	47/126 in R vs. 29/125 in PD	30/126 in R vs. 20/125 in PD	17/126 in R vs. 9/125 in PD	3.14 (1.01-9.74; p=0.05)	4.19 (1.18-14.8; p=0.03)
5	Batorova et al.; 2016 [21]	rFVIII (9) vs. pdFVIII (50)	6/9 in R vs. 7/50 in PD	4/9 in R vs. 4/50 in PD	2/9 in R vs. 3/50 in PD	7.15 (1.65-31.36; p=0.01)	NA
6	Blatny et al.; 2015 [22]	rFVIII (45) vs. pdFVIII (41)	22/45 in R vs. 20/41 in PD	3/45 in R vs. 6/41 in PD	2/45 in R vs. 0/41 in PD	1.07 (0.83-10.19; p=0.95)	NA
7	Gouw et al.; 2013 [24]	rFVIII (476) vs. pdFVIII (88)	145/476 in R vs. 29/88 in PD	92/476 in R vs. 21/88 in PD	NA	1.04 (0.65-1.66; p=0.87)	1.05 (0.63-1.74; p=0.85)
8	Xuan et al.; 2014 [23]	rFVIII (203) vs. pdFVIII (132)	14/203 in R vs. 19/132 in PD	9/203 in R vs. 15/132 in PD	NA	NA	NA
9	Strauss et al.; 2011 [25]	rFVIII (43) vs. pdFVIII (249)	14/43 in R vs. 22/249 in PD	14/43 in R vs. 22/249 in PD	9/43 in R vs. 0/249 in PD	3.43 (1.36-8.60; p=0.01)	NA

Association of Factor VIII Types (Recombinant and Plasma-Derived) With Overall Inhibitors Development - OR and HR

**Figure 2:**
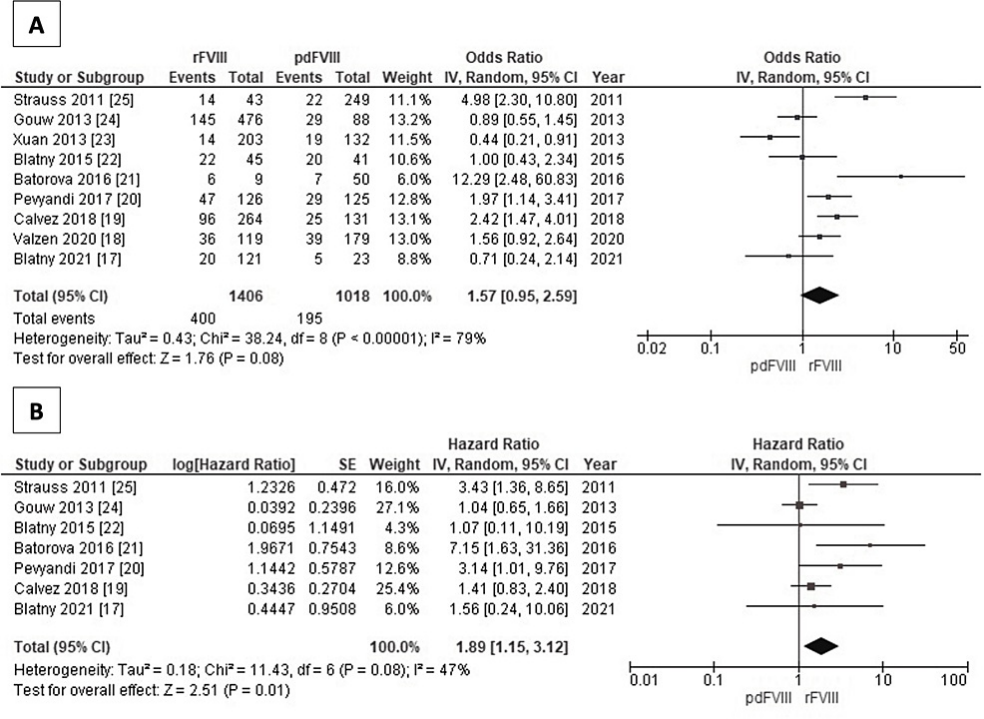
Forest plot: association of factor VIII types (recombinant and plasma-derived) with overall inhibitors development – OR (A); HR (B) The odds ratio (OR) overall inhibitor development analysis (A) used nine studies [17-25], while the hazard ratio overall inhibitor development analysis (B) used seven studies [17,19-22,24,25]. rFVIII: recombinant factor VIII; pdFVIII: plasma-derived factor VIII

Figure 2 shows two forest plots depicting the association between FVIII types and overall inhibitor development based on pooled OR and HR. All studies were included to obtain overall inhibitor development, with a pooled OR of 1.57 for rFVIII (95% CI: 0.95-2.59). There was a significant heterogeneity as shown by an I^2^ value of 79% (Figure 2A). Furthermore, the pooled HR from seven studies that provided necessary data also showed the same trend supporting higher inhibitor development even significantly in the recombinant group compared to pdFVIII (pooled HR=1.89, 95% CI: 1.15 to 3.12). Otherwise, this I^2^ test showed a smaller value compared to the previous and implies moderate heterogeneity (I^2^=47%) (Figure 2B).

Association of Factor VIII Types (Recombinant and Plasma-Derived) With High-Responding Inhibitors Development - OR and HR

**Figure 3:**
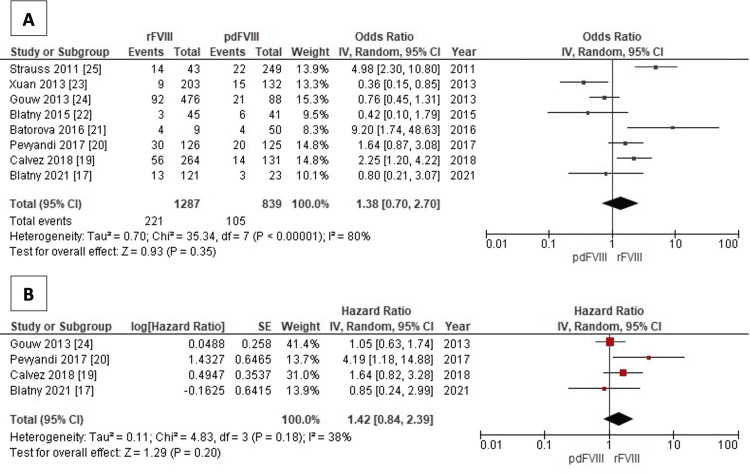
Forest plot: association of factor VIII types (recombinant and plasma-derived) with high-responding inhibitors development – OR (A); HR (B) The odds ratio (OR) overall inhibitor development analysis (A) used eight studies [17,19-25], while the hazard ratio (HR) overall inhibitor development analysis (B) used four studies [17,19,20,24]. rFVIII: recombinant factor VIII; pdFVIII: plasma-derived factor VIII

The forest plots illustrating the association between FVIII types and high-responding inhibitor development are shown in Figure 3. A total of eight studies reporting the related data were included, showing a pooled OR of 1.38 insignificantly for recombinant one (95% CI: 0.70-2.70). The heterogeneity test was significant with an I^2^ value of 80% (Figure 3A). Besides, the second forest plot consists of four studies that reported HR, supporting the previous result with the pooled value of 1.42 (95% CI: 0.84-2.39). In contrast, the I^2^ test reports a homogeneity result with a value of 38% (Figure 3B).

Discussion

Hemophilia and Available Treatments

Hemophilia A is the most common hereditary (X-linked) disorder and it occurs in one per 5,000 males worldwide [26]. Patients usually present with bleeding, but laboratory examination reveals isolated FVIII deficiency [27]. FVIII is a nonenzymatic cofactor that is needed to activate FIXa and FX respectively. This factor will trigger the conversion of prothrombin to thrombin and will directly convert fibrinogen to fibrin, as the major blood clots component [28]. The primary management of hemophilia A involves FVIII concentrated infusions. Both pdFVIII and rFVIII concentrates are available treatment options. rFVIII can be further divided into first, second, third, and fourth-generation [29]. Exposure to each type could be a risk factor for inhibitor formation due to the body's immunological response [30].

rFVIII vs. pdFVIII: HR and OR

Our pooled OR results showed a value of 1.57 for rFVIII (95% CI: 0.95-2.59). This contrasts with the findings of a previous systematic review by Franchini et al. [31], which reported slightly higher cumulative rates of inhibitor in rFVIII (CR=0.29, 95% CI: 0.26-0.32) compared to pdFVIII (CR=0.23, 95% CI: 0.15-0.33). Their study analyzed 28 prospective cohort studies involving 1,421 patients; most of them were one-arm studies or lacked comparative analyses. Besides, they also found a similar HR result between both products (adjusted HR=0.96, 95% CI: 0.62-1.49). However, our study recorded the same trend but with a significant value (pooled HR=1.89, 95% CI: 1.15-3.12). The study did not find any significant difference among the inhibitors of three generations available in recombinant groups.

Inhibitor’s development is further classified into high- or low-responding inhibitors based on patients’ anti-FVIII reaction after exposure to treatment [32,33]. According to the International Society on Thrombosis and Hemostasis Scientific and Standardization Committee, the cut-off point is 5 Bayesian Units (BU)/mL after repeated challenges with FVIII. A patient with an inhibitor value above the cut-off point is considered to have a high-responding inhibitor and vice versa. High-responding inhibitors are associated with higher cost, longer hospitalization, higher morbidity and mortality, and a greater chance of treatment failure [34].

Additionally, our study reviewed the HR and OR in high-responding inhibitors. Our results support the findings from Iorio et al. who found a greater incidence rate for rFVIII [17.4% (14.2-21.2)] compared to pdFVIII [9.3% (6.2-13.7)] [10]. This study consisted of 2,049 patients and was conducted to evaluate the incidence rates of inhibitor development in previously untreated hemophilia A patients. Of note, the HR from our study endorsed these results.

Mechanism of Inhibitor Development in Both Treatments

Inhibitors are neutralizing antibodies that bind the non-functional epitopes of FVIII, which leads to the inactivation of the product. Multifactorial risk factors, including genetic and environmental, are shown to affect the development of inhibitors. Nonsense mutations or large deletions in the FVIII gene are strongly related to the condition [35]. The development of inhibitors from the environment, one of which involves the drug used, usually involves a complex immune mechanism. During the factor injection, antigen-presenting cells will capture and present the antigen-derived peptides to the CD4+ T cell via HLA class II molecules. This T cell becomes activated and is able to stimulate B cells to become plasma cells and produce antibodies. As the usual immune response mechanism, this process will require the second trigger to produce more. The main neutralizing antibodies are IgG1 and IgG4 subtypes [36].

The result was confirmed by Whelan et al. who found a significant difference in the value of both IgG subclasses (IgG1 and IgG4) in inhibitor patients [37]. IgG4 was even completely not found in healthy subjects. Besides, our finding correlates with an experimental study in mice conducted by Delignat et al., which found that IgG4 titers were 2.4-3.2-fold higher in mice treated with rFVIII compared to pdFVIII.

Implications in Clinical Practice

This updated systematic review emphasizes the benefits of using pdFVIII compared to rFVIII for more favorable outcomes. In daily practice, a doctor will likely have to choose between both treatment options. We recommend evaluating a patient’s risk factor in developing inhibitors first before choosing the appropriate treatment. Hence, patients with high-risk factors should not receive rFVIII products. Both the risks and benefits of each drug should be considered equally.

Limitations

We acknowledge that this study has several limitations. Firstly, all included studies showed variations in the duration of the recruitment period, with the earliest point dating as far back as 1984 and the latest being 2016. Therefore, the results of the later studies may have been influenced by the availability of advanced drugs. Besides, most studies also did not provide drug subclasses used in the treatment. In addition, not all studies reported the complete data that was needed for this review.

Despite these limitations, this study has some key strengths as well. Firstly, we found that the majority of included studies had a good score in terms of bias assessment. Besides, most of the studies were cohorts, case-control, and RCTs, which are very capable of evaluating long-term inhibitor effects in patients. Another strength is the representation of a large number of countries in the studies, which made the data wide-ranging, extensive, and global in nature.

## Conclusions

This comprehensive meta-analysis demonstrates that rFVIII is more likely to cause inhibitor development, either overall or high-responding, compared to pdFVIII.

## Appendices

The search strategy and keywords used for each database can be seen in Table 3. Each keyword was arranged using Boolean Operators to conduct a comprehensive search process.

**Table 3:** Keywords or queries used in each database for the literature search process

Database	Queries	Hits
PubMed	((plasma derived AND recombinant) AND ("Factor VIII"[Mesh])) 280 AND (inhibitor) AND ("Hemophilia A"[Mesh])	280
Scopus	TITLE-ABS-KEY ((plasma derived AND “recombinant”) AND (“Factor VIII”) AND (inhibitor) AND (“Hemophilia A”))	283
ScienceDirect	(plasma derived AND “recombinant”) AND (“Factor VIII”) AND (inhibitor) AND (“Hemophilia A”)	32

The risk of bias assessment using the Newcastle-Ottawa Quality Assessment Scale was conducted on all included studies with cohort and case-control study design, which is illustrated in Table 4 and Table 5.

**Table 4:** Newcastle-Ottawa Quality Assessment Scale (cohort studies)

Studies	Selection	Comparability	Outcome	Total
Blatny et al.; 2021 [17]	****	-*	***	8 - Good
van Velzen et al.; 2020 [18]	***-	-*	***	7 - Good
Calvez et al.; 2018 [19]	****	**	***	9 - Good
Batorova et al.; 2016 [21]	****	-*	***	8 - Good
Blatny et al.; 2015 [22]	****	**	***	9 - Good
Gouw et al.; 2013 [24]	****	-*	***	8 - Good
Xuan et al.; 2014 [23]	-***	*-	***	7 - Good
Strauss et al.; 2011 [25]	****	--	***	7 - Good

**Table 5:** Newcastle-Ottawa Quality Assessment Scale (case-control study)

Study	Selection	Comparability	Outcome	Total
van Velzen et al.; 2020 [18]	***-	-*	***	7 - Good

Meanwhile, the risk of bias assessment in the randomized controlled study was conducted with the Cochrane Risk of Bias for Randomized Clinical Trial Study, the result of which could be seen in Table 6.

**Table 6:** Cochrane Risk of Bias for Randomized Clinical Trial Study + (low risk of bias); - (high risk of bias); ? (unclear risk of bias)

Study	Random sequence generation	Allocation concealment	Blinding of participants and personnel	Blinding of outcome assessment	Incomplete outcome data	Selective reporting	Other bias
Pevyandi et al.; 2018 [20]	+	?	-	-	+	+	+
